# Cytological Diagnosis of Small Cell Carcinoma of Urinary Bladder in a Patient with CLL

**Published:** 2014-02-03

**Authors:** Gülçin Güler Şimşek, Servet Güreşçi, Ural Oğuz, Ali Ünsal

**Affiliations:** 1 Department of Pathology, Kecioren Training and Research Hospital, Ankara, Turkey; 2 Department of Urology, Kecioren Training and Research Hospital, Ankara, Turkey

**Keywords:** Small Cell Carcinoma, Bladder, Cytology

## Abstract

Small cell carcinoma of the urinary bladder (SCCUB) is an extremely rare bladder malignancy characterized by an aggressive clinical behavior. So, it is important to diagnose this high grade disease by urinary cytology. We report a case of SCCUB in an old man with chronic lymphocytic leukemia (CLL) in remission, while bladder tumor was diagnosed by cytology. With this article, we aimed to review and to update the literature concerning this tumor.

## Introduction

The small cell carcinoma of the urinary bladder (SCCUB) is a neuroendocrine malignancy derived from the urothelium which mimics its pulmonary counterpart histologically (1).

SCCUB is an extremely rare bladder malignancy with a mean frequency of less than 0.5% mentioned in the literatures of Europe and of North America, and characterized by an aggressive clinical behavior (2). The disease usually occurs in male adults, with a mean male to female ratio equal to 5:1. Most patients are in the sixth to seventh decades of life. Investigators debate the origin of tumor whether it is derived from a multipotent stem cell of the bladder urothelium (3).

Treatment is primarily surgical in contrast to pulmonary counterpart. Its prognosis is generally poor even with aggressive therapy. It should be suspected clinically in patients with the complaint of hematuria. Differential diagnosis is important in the first step of urine cytology and includes metastatic SCC, high grade transitional cell carcinoma, and malignant lymphoma (4).

Chronic lymphocytic leukemia (CLL) is a monoclonal B-cell disorder that is rare as a primary lymphoma of the bladder, whereas it mostly occurs as the part of widespread metastases of hematological presentaion in systemic diseases.

## Case report

**Fig 1:**
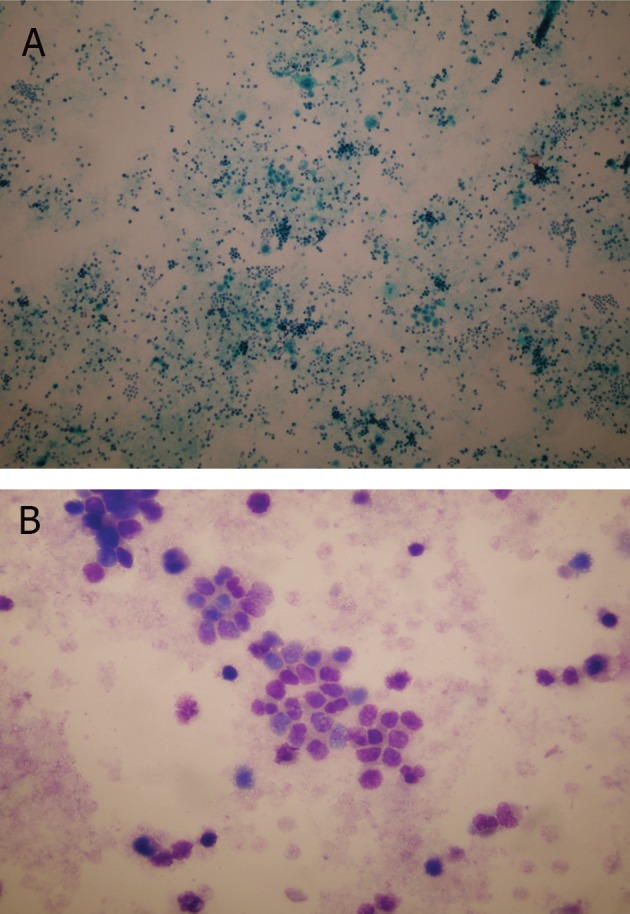
A. In the cytological examination of the urine, there are many small hyperchromatical cells similar to lymphocytes (PAP; ×40). B. Tumor cells are mostly isolated or form small clusters containing focal cell molding (MGG; ×200).

**Fig 2:**
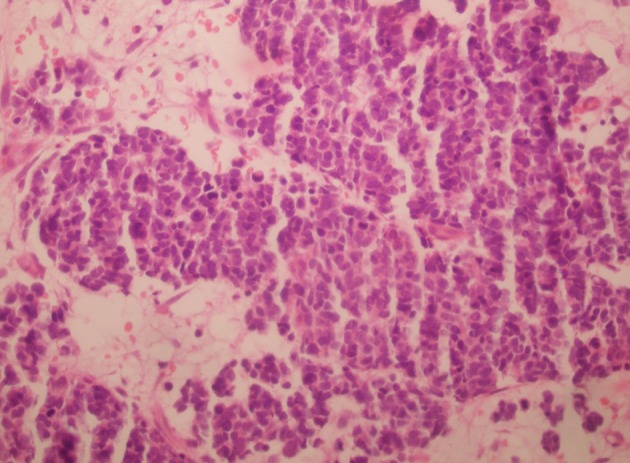
Light microscopic examination shows small hyperchromatical tumor cell sheets invading sub-mucosa and muscularis mucosa (H&E; ×100).

**Fig 3:**
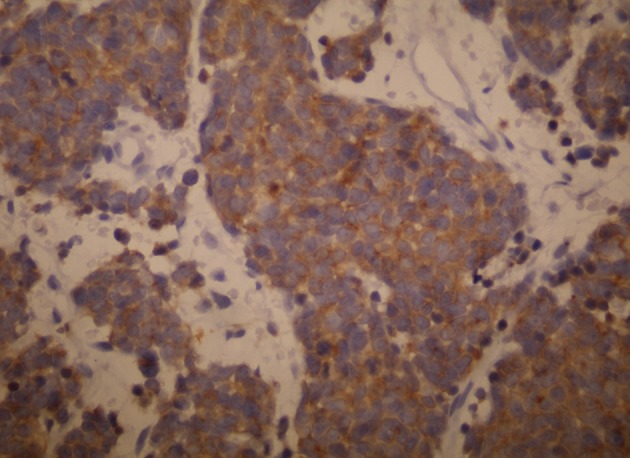
The tumor cells express pancytokeratin and neuroendocrine markers (Kromogranin; ×100).

**Fig 4:**
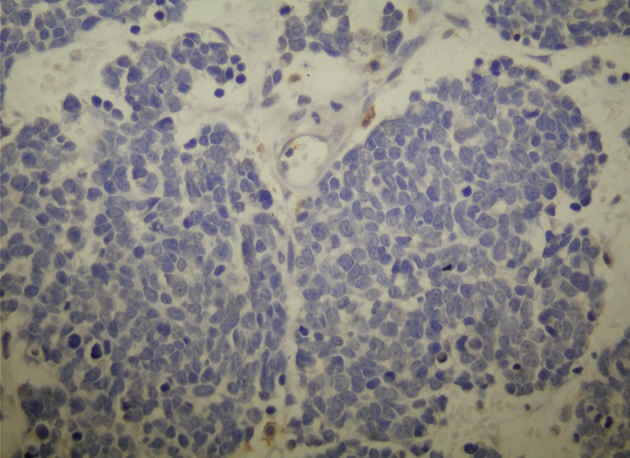
The tumor cells are negative for leukocyte common antigen (LCA; ×100).

A 82-year-old man who was diagnosed with CLL five years ago due to high white blood cells (WBC) count in complete blood and bone marrow biopsy findings. During his follow-up period, he had no symptoms until he presented at our institute, Kecioren Research and Training Hospital, Ankara, Turkey, with hematuria. There was no history of chemotherapy or radiotherapy. In the abdominal magnetic resonance imaging (MRI) examination, there was 41×42 mms polypoid mass in the anterior wall of the urinary bladder. Bone structures were normal, radiologically. After collecting the patient’s urine samples, three slides were prepared using cytocentrifuge technique, and they were then stained with May-Grünwald-Giemsa (MGG, Merck, Germany), Papanicolaou (PAP; Biostain, UK) and hematoxylin and eosin (H&E, Biostain, UK) methods. After the examination by a light microscope, many small hyperchromatical cells similar to lymphocytes were detected (Fig 1A), while they were mostly isolated or formed small groups. Focal cell molding was also noted in clusters (Fig 1B). We reported it as malignant cells in urine cytology, and the cytomorphological pattern was interpreted as small and blue round cell tumor that was consisted with SCC, but due to degenerated and separated distribution of cells in urine, differential diagnosis was difficult. Transurethral resection (TUR) was applied to the tumor. Light microscopic examination of collected samples showed small hyperchromatical tumor cell sheets invading submucosa and muscularis mucosa (Fig 2). Immunohistochemical analysis showed that the tumor cells expressed pancytokeratin and neuroendocrine markers such as synaptophysin and chromogranin (Fig 3). They were negative for cytokeratin 20 and leukocyte common antigen (LCA, Fig 4). To consider the patient’s age, no additional aggressive surgery was performed. There is no evidence of metastasis in MRI results. He was well without any evidence of recurrence 6 months after surgery.

## Discussion

SCCUB is a rare tumor which is histologically identical to pulmonary small cell undifferentiated carcinoma (5). In most cases, it occurs with other types of urinary bladder carcinomas (6), but in our case, it was pure small cell type. The clinical features of SCCUB are similar to those of transitional cell carcinoma and related to the presence of a mass. Gross hematuria is the most common symptom.

Our patient fulfiled all the histomorphological criteria of SCC, described by World Health Organisation (WHO). It consists of cells having scant cytoplasma, hyperchromatic, granular dense chromatin, inconspicuous or invisible nucleoli and round-oval nuclei. Furthermore, SCC can be differentiated from several other cancers as follows: i. direct invasion of the urinary bladder by prostate SCC, ii. metastatic SCC from other tissues, e.g. from the lung. Metastatic SCC can not be differentiated from a primary bladder SCC in the microscopical examination, iii. primary lymphoma of the bladder which mostly occurs as the part of widespread metastases of hematological presentation in systemic diseases, and iv. high grade transitional cell carcinoma (7-9).

In terms of our case, involvement of urinary bladder by hematological disease was the problem. Differential diagnosis was important due to different treatment modalities. Immunohistochemistry was extremely helpful in establishing the diagnosis. The role of molecular biology has not yet been defined.

CLL is a monoclonal B-cell disorder that is characterized by weak surface immunoglobulin heavy and light chain. The diagnosis of CLL is usually established when there is a persistent absolute lymphocytosis in excess of 10×10^9^/l. Marrow biopsy of CLL shows a diffuse pattern of involvement. Clinical progression is usually silent with a few mild symptoms. Our patient was untreated with only periodical followed-up for CLL. To our knowledge, this is the first case of SCCUB in a patient with CLL.

Isolated urinary bladder small cell neuroendocrine carcinoma (NEC) is extremely rare in immunocompetent individuals (5). However, urinary bladder can also be the site of this rare event.

Pedersen-Bjergaard et al. reported a case of carcinoma of the urinary bladder after treatment with cyclophosphamide for non-hodgkin’s lymphoma (10). Our case didn’t receive any treatment for CLL.

Jazaerly et al. reported small cell carcinoma of the ovary presenting in a urine cytology specimen (11). In this, the morphologic and immunohistochemical features of the tumor were mostly consistent with urinary bladder involvement by pulmonary-type primary ovarian.

In another article, Grignon et al. reported the clinicopathologic findings in a series of 22 cases (12). Immunohistochemical examination of abovementioned study showed positive results for neuron- specific enolase in all of cases, while the presences of cytokeratin, chromogranin, serotonin and S-100 protein were only confirmed in some of the cases. In five of seven cases, neuroendocrine differentiation was confirmed by microscopic study. Treatment and follow-up data were reported for 19 patients as follows: 10 (52.6%) were dead of the disease, 5 (26.3%) were alive without the disease, 3 (15.8%) were alive with the disease, and 1 (5.3%) died of irrelevant disease. Although overall survival was poor, some cases responded well to therapy. Radical cystectomy with adjuvant chemotherapy appears to be the treatment of choice, but there is no standard approach to the management of SCCUB.

Kibar et al. reviewed five consecutive patients with small lymphocytic lymphoma (SLL) of the urinary bladder receiving treatment in their clinic (13). The management that seems to give a better survival is the combination of radical cystectomy and chemotherapy.

The clinical outcome is poor as compared with poorly differentiated transitional cell carcinoma, and is similar to that of pulmonary small cell undifferentiated carcinoma. Neuroendocrine differentiation doesn’t make a sense in terms of aggressive behavior. It is noted that the origin of multipotent mucosal stem cell is predictable in pathogenesis (14). It is thought that smoking is a possible etiological factor (5).

Cytological differential diagnosis of this type of tumors is difficult because cell degeneration is prominent in urine specimen, but it is very important to have a quick and accurate diagnosis of SCC (15). Our case is the first one to show simultaneous presence of two forms of cancer, CLL and SCC.

## Conclusion

We would like to emphasize that small cell carcinoma should be considered in the differential diagnosis of urinary bladder masses, particularly in patients with CLL who have hematuria. Cytological differential diagnosis from the lymphoma is necessary due to different treatment modalities.
